# “*We don’t talk about these things*”: Asian American Veterans’ lived experiences and perspectives of suicide risk and prevention

**DOI:** 10.3389/fpsyt.2025.1520980

**Published:** 2025-06-03

**Authors:** Lauren S. Krishnamurti, Joseph Mignogna, Christe’An D. Iglesias, Carly M. Rohs, Evan R. Polzer, Ryan Holliday, Gayle Y. Iwamasa, Lindsey L. Monteith

**Affiliations:** ^1^ Rocky Mountain Mental Illness Research, Education and Clinical Center for Suicide Prevention, Veterans Health Administration, Aurora, CO, United States; ^2^ Department of Physical Medicine and Rehabilitation, University of Colorado Anschutz Medical Campus, Aurora, CO, United States; ^3^ Spark M. Matsunaga VA Medical Center, VA Pacific Islands Healthcare System, Honolulu, HI, United States; ^4^ Department of Psychiatry, University of Colorado Anschutz Medical Campus, Aurora, CO, United States; ^5^ Office of Mental Health, Veterans Health Administration, Washington, DC, United States

**Keywords:** Veteran, Asian American, sociocultural beliefs, suicide prevention, cultural psychiatry

## Abstract

**Background:**

Suicide rates have increased substantially among Asian American, Native Hawaiian, and Pacific Islander Veterans. However, little is known about the context of suicide risk or how best to tailor care for this population, especially as it relates specifically to Asian American Veterans, for whom extant research has been limited. We explored Asian American Veterans’ lived experiences with suicidal thoughts and behaviors, their perspectives regarding suicide risk and prevention, and the broader context in which these occurred.

**Methods:**

Qualitative interviews were conducted in 2022–2023 with 20 Asian American Veterans with histories of suicidal ideation and/or suicide attempt(s). Interviews explored participants’ identities and values (i.e., for context), as well as their beliefs, experiences and perspectives regarding suicide prevention (e.g., how suicide is discussed among Asian American Veterans, factors that might cause Asian American Veterans to experience suicidal ideation, and how suicide prevention initiatives can be tailored to address the needs of Asian American Veterans). Interview transcripts were analyzed through inductive thematic content analysis.

**Results:**

We identified four themes relevant to Asian American Veterans’ experiences with suicidal thoughts and behaviors, perspectives on suicide risk and prevention, and the context in which these occurred. First, participants spoke to the “model minority” stereotype and pressure to convey self-discipline and perfectionism and to acculturate to American or Western values, which were also shaped by their own or familial immigration experiences. Second, participants reflected upon their minoritized status within the U.S. military, which often mirrored the sense of “otherness” experienced outside of their military service. While participants described positive overall experiences in the military, they also described experiencing racism during their military service. Third, mental health stigma was salient, shaped by cultural beliefs and perceived as consistent with military culture, deterring disclosure and help-seeking and posing an obstacle for suicide prevention. Lastly, destigmatizing mental healthcare and increasing the visibility of Asian American Veterans seeking mental health services were considered paramount to suicide prevention.

**Conclusion:**

Considerations for preventing suicide among Asian American Veterans include addressing the sociocultural factors that shape mental health, suicide risk, and healthcare use. In particular, findings suggest the importance of addressing stereotypes about Asian Americans (e.g., model minority myth), preventing behaviors that contribute to a sense of “otherness,” and increasing the visibility of Asian American Veterans in outreach and messaging to promote help-seeking.

## Introduction

Suicide prevention among U.S. Veterans remains a priority within the U.S. Department of Veterans Affairs (VA), given increased suicide rates among Veterans relative to the non-Veteran adult population (1). Additionally, specific Veteran subgroups have experienced particularly concerning changes in their rates of suicide over time (2). In recognition of these divergent trends, there has been a call to tailor suicide prevention approaches to meet the “unique needs and contexts” of different groups (3).

One Veteran subgroup that has experienced a substantial increase in suicide rates over time is Asian American, Native Hawaiian, and Pacific Islander (AANHPI) Veterans. The suicide rate among AANHPI Veterans increased by 192.6% between 2001 and 2021; this increase was higher than the increases reported for other groups of Veterans, which ranged from 48.7% for Black Veterans to 78.8% among American Indian/Alaskan Native Veterans (2). While a few recent studies have reported on suicide and suicide attempts among AANHPI Veterans (e.g., 4–6), such research has historically been limited, despite calls to remedy this gap (7).

In addition, an important limitation of research to date on AANHPI Veterans has been the inability to disaggregate data despite heterogeneity between different AANHPI groups (8). In the U.S, suicide rates differ substantially between Asians and Native Hawaiian/Pacific Islanders, who had age-adjusted rates of 6.8 and 12.6 per 100,000, respectively, in 2021 (9). Among Asian Americans, suicide rates also differ when disaggregated by ethnicity—for example, suicide rates are higher among Japanese Americans (10). In a secondary analysis of post-9/11 Veterans, the prevalence of suicidal ideation and suicide attempt between different groups of AANHPI Veterans was also highly variable, with lifetime estimates of suicidal ideation ranging from 19.3% (95% Confidence Interval [CI]: 13.3, 25.3) among Filipino Veterans to 43.9% (95% CI: 13.0, 74.8) among Guamanian/CHamoru Veterans (11). These findings underscore the importance of disaggregating between AANHPI groups, when possible.

Additionally, prior research has found that Asian Americans are less likely to utilize mental health services, oftentimes at rates that rank amongst the lowest of all racial/ethnic groups (12, 13). Various systemic and cultural barriers impact Asian Americans’ use of mental health services (14), including mental health stigma, cultural beliefs about help-seeking (15), and traditional Asian values that emphasize emotional self-control, collectivism, and conformity (16). Further contributing to such stigma is the “model minority” stereotype, wherein individuals may be reticent to use healthcare services when experiencing mental distress (17–19). Due to some of these societal pressures and cultural beliefs, suicidality among Asian Americans may be “hidden” or underreported (20).

Regarding Asian American Veterans, prior studies suggest Asian American Veterans may be more reticent to engage in mental health services and may be less likely to use VA and non-VA mental health services, compared to Veterans of other racial and ethnic groups (21, 22), although these findings have not been uniform across studies. Studies have also noted the impact of race-based discrimination, including unit-based discrimination, on the mental health of Asian American Veterans and service members (23, 24). Race-related PTSD has been reported among Asian American Veterans who served during the Vietnam War (25, 26).

Nonetheless, knowledge has been limited regarding the context of suicide risk among Asian American Veterans, as well as concerning considerations for preventing suicide among Asian American Veterans. Understanding the broader context of suicide risk among Asian American Veterans is integral to understanding how to tailor suicide prevention approaches for this population. One important way of beginning to ascertain this information is by including Asian American Veterans with relevant lived experiences in suicide prevention research, which also may help with informing tailoring of interventional approaches (c.f. 27). We are unaware of any studies that have obtained the perspectives of Asian American Veterans on suicide prevention or explored their lived experiences with suicidal thoughts and behaviors.

Considering these knowledge gaps, the current study explored Asian American Veterans’ lived experiences with suicidal thoughts and behaviors, their perspectives regarding suicide risk and prevention, and the broader context in which these occurred.

## Methods

Semi-structured qualitative interviews were conducted with 20 Asian American identifying Veterans with self-reported histories of suicidal ideation and/or suicide attempt, who lived in the United States (excluding territories) at the time of research participation. These interviews were conducted as part of a larger project focused on suicide risk and prevention among AANHPI Veterans in which both subject matter experts (c.f. 28) and Veterans (the present study) were interviewed. Given the heterogeneity between Asian American and NHPI Veterans, as well as between Veterans residing in different regions (c.f. 4), in this manuscript, we focus specifically on findings with Asian American Veterans residing in U.S. states.

### Eligibility

Inclusion criteria for participation was as follows: self-identified as Asian American (which could be one of multiple racial identities); previously served on active duty in the U.S. military; had experienced lifetime suicidal ideation (i.e., thoughts of killing oneself at any point in one’s lifetime) with or without suicide attempt(s) (i.e., an attempt to kill oneself with at least some intent to die); currently resided in a U.S. state; and were 18–89 years of age. Individuals were excluded if they were currently on active duty, mobilized on Reserve or National Guard duty, or unable to provide informed consent.

### Recruitment

Recruitment efforts consisted of mailing invitation letters to AANHPI Veterans residing in U.S. states, who were identified from three different sources: USVETS (a dataset that includes Veterans nationally, irrespective of whether they have used VA healthcare); a local research repository; and among Veterans who had consented to be contacted about future research opportunities when they participated in a national survey of Veterans (ASCEND; 29). Additionally, recruitment flyers were shared with VA healthcare providers, on social media, and with members of the project’s Asian American Veteran Engagement Group (c.f. 30), comprised of Asian American Veterans with different lived experiences. Snowball sampling methods were also used for recruitment; specifically, participants were asked at the end of completed interviews to consider sharing a study flyer with other Asian American Veterans. Recruitment efforts continued until saturation was deemed to be reached and responses came to halt.

### Procedures

Individuals interested in participating contacted study personnel and were screened for eligibility. Eligible participants were scheduled for a virtual (Microsoft Teams) or phone appointment. Informed consent was obtained verbally at the commencement of the appointment. Following consent, the University of Washington Risk Assessment Protocol (UWRAP; 31) was administered to assess safety and appropriateness to participate (i.e., not at high acute risk for suicide or self-harm). Participants then answered questions regarding their demographics, military service, suicidal ideation, and suicide attempt, to confirm their eligibility to participate and to characterize the sample. Suicidal ideation and attempt history were assessed through an abbreviated version of the Self-Injurious Thoughts and Behaviors Interview (SITBI; 32), with questions added regarding timing of suicidal ideation and attempt relative to military service. Subsequently, staff trained in qualitative data collection (CDI, CR, JM, and LSK) conducted the qualitative interview, using a semi-structured interview guide created by the study team and revised with input from the project-specific Veteran Engagement Group. Veteran Engagement Group members’ input included suggestions to structure the interview holistically, such that participants could share what was meaningful to them and how those values were shaped. This allowed participants to more holistically explain their experiences. Thus, interviews explored participants’ identities, values, communities, military service, and experiences as Veterans, to provide contextual information for the subsequent questions, which queried participants’ experiences with suicidal thoughts and behaviors (e.g., precipitants, disclosure, help-seeking), and perspectives and experiences regarding suicide prevention (e.g., preferences, beliefs about what would be helpful). Interviewees were prompted to discuss what factors might cause Asian American Veterans to experience suicidal ideation, how suicide is discussed in this population, and how/where suicide prevention initiatives might be tailored to suit the needs of Asian American Veterans. Interviews were audio-recorded and professionally transcribed. On average, interviews were 42 minutes long (median: 42 minutes; range = 19, 81 minutes). Following the interview, additional self-report questions were administered (e.g., regarding military service and healthcare use) to describe the sample. The study visit concluded with the UWRAP post-assessment and debriefing. Compensation of $70 was provided for participation. Study procedures were approved by the local Institutional Review Board.

### Analysis

Four trained qualitative analysts (LSK, CR, CDI, JM) conducted an inductive thematic content analysis of interview transcripts. Backgrounds of the interviewers and analysts included clinical psychology, counselling psychology, health anthropology, and public health. All members of the team had prior experience conducting qualitative research. The team initially completed a bracketing exercise (33) to identify potential biases prior to analysis. All four analysts familiarized with a subset of transcripts and utilized an inductive approach to create an initial codebook. A code hierarchy was created using the interview guide and was piloted by all analysts for two transcripts. The team met for consensus, edited the codebook, and piloted the revised codebook on two more transcripts. After the revised codebook was finalized, the remaining transcripts were divided amongst the four analysts. Each transcript was double coded utilizing Atlas.Ti (v24). Throughout analysis, the team met routinely to reconcile differences and to adapt the codebook as necessary. After analysis was complete, the research team met with the project’s Asian American Veteran Engagement Group members to share the findings and elicit their input. The quotes that follow include participants’ self-identified ethnicity and gender to provide additional context; additional information (e.g., age, service era) was considered in analysis, but is not included with the quotes, in order to protect the identities of participants.

### Participants

A total of 56 individuals contacted our team to express interest in participating. Of those, 45 (80.4%) individuals were able to be contacted for screening, 25 (55.6%) of whom were eligible to participate. Of those ineligible at screening (*n* = 20), nearly all were ineligible because they reported no history of suicidal ideation or attempt (*n* = 19; 95.0%); one additional individual (5.0%) was ineligible due to indicating that they were not Asian American. Of those eligible at screening (*n* = 25), 20 (80.0%) consented to participate and completed the study appointment. The remaining eligible individuals (*n* = 5; 20.0%) did not respond to rescheduling attempts (*n* = 2) or canceled (*n* = 3).

Thus, the final analytic sample included twenty Asian American Veterans, who participated in this study between October 2022 and September 2023. The primary recruitment source among participants in the final analytic sample was the USVETS mailing (*n* = 12; 60.0%), followed by the ASCEND mailing (*n* = 5; 25.0%); the remaining participants found out about the study from the repository mailing (*n* = 1; 5.0%), after participating in another research study (*n* = 1; 5.0%), or from a Veteran engagement group member (*n* = 1; 5.0%).

## Results

### Sample descriptives

Participants emphasized the importance of not being grouped together into a singular Asian American community, emphasizing the differences within this conglomerate group. Reflecting this, our sample of Asian-identifying Veterans was highly diverse (**Table 1**), comprising multiple Asian ethnicities, including Filipino, Chinese, Korean, Japanese, Thai, Vietnamese, Indian, Indonesian, and Malaysian. Additionally, while all participants identified as Asian, over one-third (35.0%) also identified as White, and a few (10.0%) identified as Hispanic. The sample was also diverse with respect to age (median = 48.5; range 25-77), education, employment, household income, rurality, and region, with the West the most common region of residence (55.0%). The majority of participants were men (80.0%), heterosexual (90.0%), married (65.0%), and had never been homeless (85.0%). Regarding military service, the sample was diverse regarding military service type, branch, service era, deployment history, rank at separation, duration of active duty service, and time since discharge. The majority of participants (90.0%) had been honorably discharged.

**Table 1:** Sample demographics and military service characteristics (*N* = 20).

	Mean	SD
Age^a^	51.1	14.2
Duration of active duty service (years)^d^	7.5	8.3
Time since discharge (years)^e^	21.5	16.8

	n	%
Race^b^		
Asian	20	100
White	7	35
Asian Ethnicity^b^		
Filipino	7	35
Chinese	5	25
Korean	2	10
Japanese	2	10
Thai	2	10
Vietnamese	2	10
Indian	1	5
Indonesian	1	5
Malaysian	1	5
Hispanic Ethnicity		
Non-Hispanic	18	90
Hispanic	2	10
Gender		
Men	16	80
Women	4	20
Sexual Orientation		
Heterosexual	18	90
Gay, lesbian, bisexual, questioning, or other	2	10
Marital Status		
Married	13	65
Separated/divorced	4	20
Never married	2	10
Widowed	1	5
Education		
High school	3	15
Some college or Associate’s degree	5	25
Bachelor’s degree	7	35
Master’s or doctoral degree	5	25
Employment		
Working	11	55
Not working, retired	7	35
Not working, disabled	2	10
Household income		
<$12,500-$34,999	3	15
$35,000-$59,999	4	20
$60,000-$99,999	4	20
$100,000 or more	9	45
Homelessness		
No, never	17	85
Yes, previously or currently	3	15
Region of residence^c^		
West	11	55
South	4	20
Midwest	1	5
Northeast	4	20
Rurality		
Small town	8	40
Medium-sized town	2	10
Suburb	7	35
City	3	15
Military service^b^		
Active duty	20	100
Reserve duty	7	35
Branch of service^b^		
Army	11	55
Navy	5	25
Air Force	5	25
Marine Corps/Coast Guard	2	10
Service Era^b^		
OEF/OIF/OND	13	65
Desert Storm/Desert Shield	6	30
Post-Vietnam/Peacetime	6	30
Vietnam	2	10
Post-Korean War	1	5
Deployed		
No	7	35
Yes	13	65
Rank at Separation		
Enlisted, E1-E3	2	10
Enlisted, E4 or higher	10	50
Non-Commissioned Officer	3	15
Warrant Officer	2	10
Officer	3	15
Discharge type		
Honorable	18	90
Other^f^	2	10

OEF, Operation Enduring Freedom; OIF, Operation Iraqi Freedom; OND, Operation New Dawn.

a Median = 48.5, range = 25-77.

b Not mutually exclusive (i.e., participants could report multiple response options); thus, total percentage exceeds 100%.

c Based on U.S. Census region.

d Median = 4, range = 1-30.

e Median = 17, range = 3-57.

f Included general (*n* = 1) and missing (*n* = 1).

Most participants had a VA service-connected disability rating (75.0%) and reported that they had used Veterans Health Administration (VHA) services (75.0%) (**Table 2**). Most (80.0%) indicated that they felt that they had needed professional care for their mental health care in their lifetime, and most (80.0%) also indicated that they had received professional care for mental health care at some point. The vast majority (85.0%) of participants also reported experiencing lifetime trauma exposure. All participants reported lifetime suicidal ideation, over half (55.0%) during their military service, and nearly all (95.0%) at some point following their separation from the military. One-fourth (25.0%) reported a lifetime suicide attempt.

**Table 2:** Healthcare utilization, suicidal ideation, and suicide attempt (N = 20).

	*n*	%
**VA service-connected disability rating**	15	75
**Use of services provided by VHA**	15	75
**Ever *needed* professional care for mental health** ^a^	16	80
**Ever *received* professional care for mental health** ^b^	16	80
**Lifetime trauma exposure** ^c^	17	85
**Lifetime suicidal ideation**	20	100
During military service	11	55
Following military service	19	95
**Lifetime suicide attempt(s)**	5	25
During military service	2	10
Following military service	3	15

VA, Department of Veterans Affairs; VHA, Veterans Health Administration.

a In the full sample, when asked if they had ever *needed* professional care for a mental health or substance use problem, 35.0% of participants (*n* = 7) indicated that they had needed such care prior to leaving active duty, and 65.0% of participants (*n* = 13) indicated that they needed such care after leaving active duty. Responses were not mutually exclusive.

b In the full sample, when asked if they had ever *received* professional care for a mental health or substance use problem, 30.0% of participants (*n* = 6) indicated that they had received such care prior to leaving active duty, and 70.0% of participants (*n* = 14) indicated that they received such care after leaving active duty. Responses were not mutually exclusive.

c Assessed through the first question of the Primary Care PTSD Screen for DSM-5 (PC-PTSD-5; 34).

### Qualitative interview themes

In analyzing qualitative interview transcripts, we identified four themes. The first involved the immigration-related experiences of Asian American Veterans within this sample, the “model minority” stereotype, and pressure to convey self-discipline, perfectionism, and to acculturate to American and Western values. The second theme encompassed Veterans’ experiences serving in the U.S. military, which included minoritized status, a sense of “otherness,” and varying experiences of discrimination. The third theme included mental health stigma, as well as typically forgoing disclosure of suicidal ideation or help-seeking for mental health concerns. The final theme comprised recommendations for preventing suicide, which included destigmatizing mental healthcare and increasing the visibility of Asian American Veterans seeking mental health services. Next, we describe each of these themes in more detail.

### Immigration and the “model minority” stereotype: “Work hard, do everything right, and not make trouble”

Immigration (whether themselves or their parents) was a consistent theme that appeared to shape how participants discussed and viewed themselves and their families within both American society and their military careers. Participants described varying experiences that included being born in the U.S., immigrating with their families to the U.S. as children, or being adopted from outside of the U.S. As one participant shared, *“I identify as Vietnamese American. I came to the United States when I was a baby. My parents were boat people who escaped from there … my parents did not let me forget the sacrifices they made to bring me here to America so I didn’t have to grow up in Communist Vietnam. And that shaped a lot of how I grew up.”* (Vietnamese woman) Participants described feelings of not being seen as American due to perceived otherness. As one participant described, *“I immigrated to this country … when I was [a child] with my mom, dad, and my brother from China, so I’m 100% Chinese. I just think like I’m an American like a White person or a Black person, but a lot of America doesn’t see it that way.”* (Chinese American man) Similarly, another participant noted, “*People are always like ‘what are you?’ And you’re like ‘I’m an American.’*” (Korean man) Another participant described the pressure to assimilate that he felt as an immigrant: “*I feel like I’ve always had like a strong pressure to assimilate and … to minimize my otherness to the maximum extent possible. Like I don’t* sp*eak with a recognizably Asian accent … I’d say there’s almost a pressure to be as ‘normal’ and assimilate to the dominant culture to the maximum extent possible, and any deviation from that is negative and has consequences*.” (Filipino/Malaysian man)

As a result of sacrifices on behalf of their parents, participants described upbringings consistent with what has been referred to as the model minority myth. A participant stated, *“I think we tend to be perfectionists, or at least I do … We tend to want to just perform at the highest level possible.”* (Chinese man) Another participant explained that this pressure to work hard and “perform at the highest level” may be felt especially by first-generation Asian immigrants: “*My mom was very strict, Korean born, and I was Korean born, so first-generation Asian mothers are not easy. You’re just told to work hard, do everything right, and not make trouble. You know, that kind of thing*.” (Korean/multi-racial woman) Further describing the ways in which the model minority stereotype persists, participants described not wanting to stand out. A participant shared, “*Culturally, [Asian] people just try to make themselves small and not seen … like just do your job and just g*et al*ong.”* (Korean/multi-racial woman) This sentiment carried into some participants’ military service in relation to their work ethic: “*We always seemed like we stayed back, and we just conformed, and we never got in trouble … We just did what we had to do.”* (Japanese man) Some connected this pressure to conform and stay “out of trouble” with broader cultural values, emphasizing the importance of respect for family and authority across different Asian cultures: “*I think that they have strong family values and that carries on to the military, which they have a lot of respect for people in positions, elders, to respect for authority, where I felt that they are, we are usually academic, educated, usually, and really just don’t cause trouble.”* (Japanese man)

### Experiences in the U.S. Military: “*There’s not many of us”*

Experiences in the military varied widely between participants and even within individuals, with some individuals describing a range of experiences during their service. When discussing their military service history broadly, the majority of participants spoke positively, noting that serving had provided them with unique opportunities and experiences from which they grew, as well as strong friendships: “*I enjoyed my time in the military, meeting a lot of different people and just being independent and on my own.”* (Filipino/multi-racial woman) Another participant stated regarding his military service: *“I really enjoyed it. I think it helped me grow up. It helped me learn self-discipline. It helped me learn to follow a chain of command and to take orders.”* (Chinese man) Others described how shared military experiences had brought them closer to their peers and that they were able to create strong bonds because of their experience serving together: *“When you’re in the service, we’re all in this together, we’re all buddies … it was mostly based on our situation, rather than our ethnic background.”* (Filipino man)

A few participants indicated that that their racial and ethnic background did not come up often during their service, noting that their shared mission and service were more important: “*We didn’t really talk about our ethnicity … it just was a nonissue. We were there to serve as people, as individuals.*” (Vietnamese woman) Another participant stated, “*I didn’t really give a whole lot of thought to it, I mean, occasionally, it would come up, but I mean, so as long as you do your job, it didn’t really matter what your race was.*” (Filipino man) One participant described how, as a leader within his unit, his peers generally “*welcomed [him] with open arms*” (Thai/multi-racial man)

Nonetheless, participants also described awareness that they were a numerical minority within the military, noting the lack of Asian Americans within the “predominantly White” military culture: *“There’s not many of us. There’s not many Asian Americans in the military in general. I think I only saw like a handful when I was in.”* (Korean man) Another participant stated: “*Usually the majority is Caucasian, so I was the only Amerasian in the group.”* (Thai/multi-racial man) Similarly, another participant shared: “*I don’t know of any other Veterans that are Asian.*” (Japanese man)

Participants described their military experiences as often mirroring the ways in which they been “othered” in civilian society pre- and post-service. A participant shared: “*It’s not something that I often thought about, but there were times where it was like pointed out to me by others that ‘hey, you’re different.’”* (Filipino/Malaysian man) Another participant indicated, “*There’s not a whole lot of us. So when people see you, it’s like, ‘hey, look, there’s an Asian guy here for once.*” (Korean man) Another participant described feeling as if Asians are perceived as not being as patriotic as others, which he sometimes felt in the presence of his fellow service members: “*They don’t see Asian [as] the same type of like patriot, so it’s like no, Caucasian, Black, Hispanics, like when I walked into like a mission barbeque, for example … I see people that think of a military member, a patriot. Asian American is not the first thing that pops into their head*.” (Chinese man) Most participants in this sample described viewing these experiences of being “othered” during their military service as a part of their military experience, rather than as the defining experience of their service.

Participants’ experiences of being “othered” ranged from remarks about their race, which some participants described as “jokes” or as part of military culture more broadly, to less frequent, but more threatening or ostracizing experiences. Reflecting on this dynamic within the military, a participant described experiencing derogatory comments about his race as “*a little ribbing here and there, but that’s just the military in general.*” (Chinese man) Another participant stated, “*You just kind of learn how to take a joke and shit like that.*” (Korean man) Similarly, a participant stated, “*Anytime I had like anything racially, like negative, thrown at me, it was all in jest, it wasn’t in seriousness.*” (Korean man) Along these lines, another participant described, “*Like my Puerto Rican friend would … throw me some shade. I’d throw it back at him … it’s all in jest.”* (Filipino man)

Some participants described experiencing more overt racism during their military service: “*Hearing people refer to you as ‘slant eyes’ or ‘yellow skin.’ You know, derogatory terms.*” (Vietnamese man) Most experiences of overt racism described by participants appeared to occur within the context of a specific conflict. For example, one participant (an Indian man) described how he “*looked like the enemy*” while serving in Iraq, noting that this caused tension with his peers: “*I’ve even heard it from superiors. I’ve heard it from others, you know, due to my background and my skin tone, I look like the enemy to them, the terrorists, the Arab.”* He indicated that this racism faded “*once they got to know me and they knew that I’m not the enemy, I’m signing up to serve just like you, you know*.” An older participant (Japanese man) described being ominously threatened, due to his race, by a fellow service member during the Vietnam war: *“He looked at me and he says, he said, ‘don’t you wake me up now’ because he had a carbine next to his bed, and he said, ‘I might mistake you for a [deragatory racist term] then.’ A [censored] means like a Vietnamese or VC, you know, Viet Cong, the enemy.”*

### Stigma toward mental health and non-disclosure of suicidal ideation: “*We kind of keep things to ourselves”*

Participants described a pervasive stigma surrounding mental health within the broader Asian community, including within their own families. They described shame, guilt, perceptions of weakness, and an overall intolerance of publicly disclosing mental health concerns, including suicidal ideation: “*We don’t share our dirty laundry, one. And two is we don’t—we’re strong. Like we don’t need to show other people that we’re weak and we don’t need anybody else except family. Except your family’s never gonna wanna hear like you’re sad or you can’t pull yourself up by your bootstraps and pull yourself out of this funk, right? So, it’s like they handcuff us and then they also take away the key.*” (Vietnamese woman) Another participant shared: “*They don’t like to complain … don’t like to … stir the pot or rock the boat, so they won’t say too much and that’s pretty common with Asians that I’ve found, so you’re not gonna hear too much, and we kind of handle things ourselves, we kind of keep things to ourselves.”* (Japanese man) Because of cultural norms surrounding disclosure of one’s personal and mental health concerns, some participants described being silent about their own mental health struggles and refraining from asking for help: *“You didn’t talk to someone about it, or you didn’t ask for help with it, and you just dealt with it. So I think that has caused me to not reach out for any conversation, help, or guidance around that issue.*” (Vietnamese man) At times, this cultural silence around mental health also intersected with the model minority myth and the pressures of being an immigrant: “*I like to be a proud Asian American, set an example, be a community leader. I don’t want to be like a scapegoat that like Asians are weak and submissive, [that] they’re lazy … I like to prove that stereotype wrong. I don’t want to be the weak link*.” (Chinese man) This participant elaborated further: “*I think a lot of Asian Americans tend to be very prideful, so anything that causes embarrassment or shame would probably be best avoided for folks from that type of culture because that tends to be a very sensitive kind of area.*”

Relatedly, some participants indicated that it was almost unheard of to disclose suicidality to others: “*No, I always kind of kept it in*.” (Thai/multi-racial man) Some participants explained that they did not want to burden others or lacked the “type of relationship” in which they would disclose to close friends or family that they were experiencing thoughts about suicide: *“I would’ve only had my mother, father, brother, and that’s not the type of relationship we had*.” (Japanese man) For another participant, participating in the present study was the first time he had ever disclosed a history of suicidal ideation to anyone: “*I’ve never shared it.*” (Filipino man) The cultural stigma and silence surrounding mental health and suicide were described as also being consistent with military culture, persisting within this context. As one participant described, “*I think [mental health] wasn’t something we really talked about in the service … and then as part of my upbringing, I think in my experience, and I think a lot of other Asian Americans, we have immigrant parents. Right? There’s a lot of stuff that’s just not discussed … and suicide is certainly one of those things that, you know, it’s just never a topic of conversation. We don’t talk about these things.”* (Filipino/Malaysian man)

For the few participants who had disclosed suicidal ideation to others, most reported confiding in a close family member, spouse, friend, or medical professional: “*I have [talked] to friends. That’s why I haven’t done it [attempted suicide] … I’ve got some good friends, and they served too, so they’ve been very helpful*.” (Korean/multi-racial woman); “*My wife, my* sp*ouse at the time … She’s the only one that I told*.” (Vietnamese man) For participants who had disclosed to a medical professional, it typically occurred during a routine mental health screening: “*I was in a unit, and they asked. Like each year they have like a screening. And it was an RN, right, who has to ask these screening questions and she asked. Gosh, I don’t think—I think we both were surprised about like my reaction*.” (Vietnamese woman)

Although all participants reported a history of suicidal ideation, few described reaching out for help when they were suicidal, whether through the VA or elsewhere. Rather, participants described alternate ways of coping with suicidal thoughts, such as speaking with friends or others with similar histories: *“Talking to the friends that I do have, like on the phone … just able to hear voices of people you trust”* (Filipino/multi-racial man) Similarly, another participant described *“talking to people with similar backgrounds.”* (Chinese man) Others described coping by engaging in activities: *“I would go out to the lake, launch my boat, and just float out there, not even start up the engine. I’d just float out there, and I don’t know. I don’t know how to describe it other than it was just like a calm that kind of washed everything away.”* (Vietnamese man) Another participant described coping through exercise: *“My way of coping and why I think I was able to get through is through exercise.”* (Japanese/multi-racial man)

Those who sought care when they were suicidal described mixed experiences. Some discussed accessing Veteran-specific services and having positive experiences. For example, a participant shared the following about his experience with the Veterans Crisis Line (VCL): “*I’d just, you know, Facetime and talk to them. I called the VA* sp*ecial number … and I talked to a lot of really nice people, talking out of the things, like a pep talk.”* (Chinese man) Other participants described experiences that they viewed as more negative, such as a participant who felt as if his healthcare provider had relied on prescribing him medication, rather than listening to his concerns: *“[The provider] didn’t want to listen, he just prescribed me on, he put me on medication … I didn’t have a good experience with that.”* (Japanese man) Another participant described feeling comfortable talking to the VCL responder, but dissatisfied with a call being escalated: *“I was more comfortable talking to the person on the other end of the phone and sharing some of these things I’m sharing with you, you know, but they send the police [to] my house … I don’t like that.”* (Indian man)

### Enhancing suicide prevention: “Normalize the idea that there are other people out there that are hurting”

Participants had several suggestions to improve suicide prevention for Asian American Veterans. In particular, the need to erode mental health stigma and normalize mental health care broadly within the U.S. infused participants’ suggestions: “*Seeking mental health services and suffering from mental health challenges needs to be destigmatized*.” (Filipino/Malaysian man) Another participant emphasized the need to normalize seeking out mental health care: *“Just that there’s information out there that people would feel comfortable with contacting someone for help when they needed it and to help kind of normalize the idea that there are other people out there that are hurting and may need help.”* (Chinese man) Along these lines, participants also noted the importance of acknowledging the specific cultural stigma that might be felt to a greater extent among Asian American Veterans: *“Acknowledging … starting a conversation with like, ‘hey, I know it’s not like expected … maybe it wasn’t cool in the way that you were brought up to talk about mental health, but I am going to have a conversation about mental health with you and ask point blank.’”* (Vietnamese woman)

To address mental health stigma, participants encouraged starting conversations about mental health sooner rather than later, such as during military service, particularly given the potential for trauma within the broader Asian American community. For example, a participant noted the importance of *“comfort in talking about these things*,” indicating that this type of discussion *“probably has to start while we’re in the service, not after. I kind of feel like if you start after, you’re just really playing catch up because we do have some … we’re a community of people that experience some extreme things. So I think that discussion has to happen while we’re still in the service.”* (Filipino/Malaysian man) Participants also emphasized the importance of letting Veterans know, following their discharge from military service, what services exist. They emphasized that this information could be shared through multiple channels: *“I know there is an Asian Pacific Islander group in [location], and if the VA could reach out through them, letting them know about services … I just think that might help, and maybe not just through the AAPI community, maybe the Veteran Services could let folks know more in general about mental health services that Veterans can access.”* (Japanese man)

Finally, participants noted the need to increase the visibility of Asian American Veterans in mental health and suicide prevention outreach efforts and messaging: *“I just don’t recall ever seeing any type of advertisement or solicitation* sp*ecifically for the AAPI community to get help, seek help. To me, it was in general.”* (Vietnamese man) Of note, participants emphasized enhancing the visibility of the Asian American Veteran community, not just in relation to mental health, but also broadly. As one participant described, *“More commercials, more media, more coverage that Asian Americans [are] as patriotic as any American, and they serve our country just with the same pride and heart, like any other Americans, and they deserve to be treated with respect and opportunity as any other Americans. We’re no different.”* (Chinese man)

## Discussion

The present study is among the first to examine Asian American Veterans’ lived experiences with suicidal thoughts and behaviors, along with their perspectives regarding suicide prevention. Our analysis of interviews conducted with a sample of Asian American Veterans reveals several important findings, both in regard to Asian American Veterans’ perspectives and experiences pertinent to preventing suicide (e.g., cultural stigma regarding mental health and suicide), as well as regarding the broader context for these beliefs and experiences (i.e., model minority stereotype, being “othered,” and racial discrimination for some during their military service).

Stigma regarding mental health and suicide was a salient finding. While the stigma regarding mental health and suicide within Asian cultures has been well-documented (35; 14), stigma research pertaining to Asian American Veterans has been more limited. Our findings suggest that Asian American Veterans’ cultural beliefs and experiences shape their attitudes and willingness to discuss mental health concerns and suicidality, and that these, in turn, influence their attitudes toward and use of healthcare for mental health concerns and suicidality. Concerns about bringing shame upon one’s family or being viewed as weak also may be exacerbated by the pressure imposed by the model minority myth, which may be particularly salient to Veterans whose families immigrated to the U.S. more recently. The model minority myth has been well-documented as a pervasive, racist stereotype (36; 37) that, despite being discredited, has resulted in negative pressure still felt by many Asian Americans (38). These negative pressures and other potential deleterious mental health outcomes can be further heightened by personal trauma exposure, as reported by the majority of Veterans in this sample (i.e., in self-report questions), and also more broadly by familial or collective trauma that such negative stereotypes and norms may inflict on this population (39). Furthermore, military norms regarding help-seeking may further reinforce Asian American Veterans’ beliefs about not disclosing or seeking help for mental health concerns and suicidality. In the present study, stigma was described as a strong deterrent to disclosing suicidality and seeking out healthcare for mental health concerns and suicidal thoughts or behaviors, despite all participants reporting a history of suicidal ideation and/or suicide attempt.

It is also worth noting that while participants largely reported positive experiences during their military service, which encompassed career experiences as well as developing close relationships with other service members, many interviewed for this study voiced feeling a sense of “otherness,” along with pressure to adhere to stereotypes that reinforced a sense of not being a “part of the group.” While some participants reported feeling as if their race and ethnicity had minimal impact on how other service members treated them, most participants were cognizant that they were the only Asian American individuals in their units. Some also described experiencing racial discrimination, including while deployed to war zones, that included threatening remarks. Although participants did not explicitly attribute their experiences of suicidal ideation to these experiences, race-based discrimination has been associated with depression, posttraumatic stress disorder, and suicidal ideation and attempt in other samples (40). Of note, anti-Asian rhetoric and the persistence of the “model minority” stereotype in the United States has prompted calls for action in addressing discrimination against Asian Americans (41, 42). Our findings underscore the need to prevent and address racial discrimination that occurs during military service, particularly considering that internalized racism has been associated with suicidal ideation (40). Given the association between racism and suicidal ideation (e.g., 43, 44; 40), preventing and addressing racial discrimination may be an integral component of suicide prevention for Asian American Veterans.

Finally, the present study underscores the need to increase the visibility of Asian American Veterans in outreach materials and messaging, both broadly as well as specifically regarding mental health and suicide prevention. Asian American Veterans in the current study voiced not being included or represented, both in materials that included service members or Veterans broadly, as well as in materials focused on mental health and suicide prevention. Indeed, the “invisibility” of Asian Americans, which may be exacerbated by societal beliefs regarding the model minority myth, has been discussed extensively (45). Increasing the inclusion and visibility of Asian American Veterans in outreach efforts and public health campaigns that normalize help-seeking may help with decreasing the stigma surrounding mental health and suicide and increasing help-seeking in the context of such experiences. Specifically, prevention efforts may be strengthened by including images and/or language on materials that include Asian American Veterans, to convey that such resources and programming apply to them. In addition, considering cultural differences that may impact messaging for different health concerns (46, 47), finding ways to optimally tailor messaging to address cultural concerns (e.g., concerns about help-seeking bringing shame upon one’s family) and that fit with Asian American Veterans’ cultural beliefs is recommended. Messaging to address stigma and regarding mental health and suicide prevention resources that are available to Asian American Veterans should also consider using multiple messengers, both from within the Asian American community, as well as from other Veterans and Veteran-serving organizations. Finally, as mentioned by some participants in our sample, discussions of mental health and suicide prevention should begin early (e.g., during military service, rather than afterwards).

Additional considerations regarding suicide prevention for Asian American Veterans include use of a holistic approach inclusive of both cultural sensitivity and cultural humility (c.f. 28). The Asian American Veteran population reflects myriad cultures, beliefs, geographic regions, and histories, all of which may impact beliefs regarding mental health, suicide, and help-seeking, as well as considerations for preventing suicide (8). Moreover, as many Veterans voiced multiple methods of coping (e.g., exercise, social support) in the absence of seeking out mental health services, augmenting evidence-based care with holistic options may be beneficial. This was also underscored in a separate study in which subject matter experts were interviewed about considerations for suicide prevention among Asian American and Pacific Islander Veterans (c.f. 28). Nonetheless, further research pertaining to these considerations is necessary.

### Limitations

While there are strengths to this study, including the diversity of the sample with respect to demographic characteristics and military service history, it is also important to acknowledge study limitations. First, despite including Veterans of different Asian ethnicities, there is substantial heterogeneity within different Asian cultures, not all of which was necessarily reflected within our sample. Additionally, although two of the co-authors identify as Asian American, none of the interviewers or analysts identify as Asian American or as Veterans, which is a limitation that could have inadvertently impacted data collection, analysis, or interpretation. We attempted to address this, in part, by engaging in bracketing, as well as by partnering with a project-specific Veteran engagement group comprised of Asian American Veterans with different lived experiences; however, it is possible that individuals with different lived experiences (e.g., Asian American Veterans themselves) might have elicited different responses from study participants or have interpreted the qualitative findings differently. In addition, we did not systematically ask participants about their generational status within the U.S. or country of birth and thus are not able to speak to whether participants’ experiences and perspectives differed based on such characteristics; however, prior research suggests that exploring such characteristics is warranted (e.g., 48–52); as such, this remains an important area to examine in future research with Asian American Veterans. Finally, considering the substantial stigma surrounding suicide noted by participants, such concerns may have precluded more in-depth discussion of experiences of suicidality by study participants or have prevented Asian American Veterans with more pervasive concerns about stigma and suicidality from participating.

## Conclusions

Considerations for preventing suicide among Asian American Veterans include acknowledging the potential influence of sociocultural beliefs and experiences which may impact suicidality and help-seeking. In particular, cultural beliefs about mental health (e.g., stigma, appearing weak) may negatively impact disclosure and prevent help-seeking for mental health concerns and suicidality among Asian American Veterans. Societal beliefs regarding the “model minority” myth, as well as Veterans’ experiences of discrimination and being “othered,” may adversely impact mental health and suicidality within this population. Addressing the stigma regarding mental health that may occur due to cultural and military beliefs and experiences is integral to suicide prevention for these Veterans. Fostering a military culture that encourages help-seeking may be particularly salient for Asian American Veterans, considering pre-existing cultural beliefs that may prevent help-seeking. Bolstering outreach materials that are tailored to Asian American Veterans, as well as increasing the visibility and overall representation of this community, may help to normalize and increase help-seeking within this population. Integral next steps for prevention include developing such materials, and evaluating the acceptability and effectiveness of this content, as crucial components of ensuring that suicide prevention is equitable and optimal for *all* Veterans, including for Asian American Veterans.

## Data Availability

Data utilized in this study cannot be publicly shared because of privacy and confidentiality requirements. De-identified data may be available from the VA Eastern Colorado Healthcare System Research and Development Committee (telephone contact: 303-399-8020) for researchers who meet the criteria for access to confidential study data.
