# Supplementary material for: “We don’t talk about these things”: Asian American Veterans’ lived experiences and perspectives of suicide risk and prevention
Source: Front Psychiatry. 2025 Jun 3;16:1520980. doi: 10.3389/fpsyt.2025.1520980 (PMC12172623; doi:10.3389/fpsyt.2025.1520980)
Supplement: Supplementary file 1 [file SupplementaryFile1.docx]

# Supplemental Appendix A. Interview Guide

Thank you for your willingness to participate in this interview to help us learn more about your experiences as a Veteran who identifies as Asian American, Native Hawaiian, and/or Pacific Islander (AANHPI). My research team’s goal with this interview is to learn more about what places AANHPI Veterans at risk for suicide, including any experiences or factors that are unique to or particularly important for AANHPI Veterans. My research team seeks to apply that knowledge to shape suicide prevention materials and interventions for AANHPI Veterans. We are also interested in understanding if there are any important regional differences in suicide risk factors among AANHPI Veterans and considerations for suicide prevention strategies for AANHPI Veterans in [the region where you live]. Your feedback is extremely valuable, whether positive, negative, neutral, or a mix.

**Identity and military service**

To begin, can you tell me a little bit about yourself? How you identify culturally, racially, and ethnically? *[Explore their race and ethnicity and what words they would like for us to use in talking about this during the interview]*

Thank you for sharing. How do you personally define AANHPI?

How did you found out about this research study? What motivated you to participate?

Can you tell me a little about your military experience (e.g., branch served in, when, circumstances that prompted joining the military)?

What comes to mind when you think of AANHPI Veterans? What are unique strengths of AANHPI Veterans? What challenges do AANHPI Veterans experience?

**Values and community**

Where do you currently live? Is there anything that stands out to you about the region where you live (or where you have lived since your military service ended) that is important for understanding the experiences you have had?

For these next couple of questions, please think about the community that you live including those you see and talk to regularly.

What led you to this community?

(Were you raised in this community, or did you move there post-discharge?)

Who makes up your community? (Does this community include family members?)

1. Can you tell me a little about the values of your community? (For example, what is important to your community and how were those values shaped?)
2. Next I’d like to understand more about your personal values – what is important to you and how were these values shaped?
   1. Do your current values reflect the values that you were raised in?
   2. How do your personal values compare to your community’s values?

I would also like to understand how suicide is viewed in your community. [Pause for response]

Is suicide talked about in your community? If so, how is it talked about? In what contexts?

Do the community values (those you mentioned previously) influence whether and how suicide is viewed and talked about in your community?

**Military service and Veteran experiences**

What was it like to serve in the military as an AANHPI individual?

Do you feel like your racial/ethnic identity shaped your experiences in the military in any ways? If so, please describe.

What were your experiences like as an AANHPI Veteran (after your military service ended)? (For example, with respect to re-integration, benefits, use of VA or non-VA healthcare, etc.)

1. What challenges have you and other AANHPI individuals whom you know experienced [prior to, during, and following military service]?
   1. How do you think these compare to the challenges and experiences of non-AANHPI Veterans?
   2. Do you think that there are any challenges that AANHPI Veterans experience that AANHPI individuals who have not served in the military do not experience?

**Suicide among AANHPI Individuals**

We want to better understand what causes AANHPI Veterans to be suicidal - that is, what causes them to think about suicide, even if such thoughts are fleeting or there is no intent to act on such thoughts; or to act on thoughts of suicide – for example, by attempting suicide or engaging in any behaviors in preparation to do so.

1. To what extent is suicide discussed among AANHPI Veterans?
   1. If it is discussed, how is it discussed and with whom?
   2. Are there any important considerations for how to discuss suicide risk and prevention with AANHPI Veterans, including with respect to the specific words or language used?
2. What do you think needs to be done to improve suicide prevention for AANHPI Veterans?
3. Is there anything unique or important that needs to be done to prevent suicide among AANHPI Veterans, including among those in the region in which you live?
4. In what ways could current efforts be better tailored toward AANHPI Veterans? What things do you think would not be helpful? What could be improved upon?
5. In what formats and by whom should such efforts be offered or delivered?

**Personal experiences with suicidal thoughts and behaviors**

The next set of questions are about times in your life when you have experienced thoughts of suicide (such as thoughts of killing yourself, even if those thoughts were fleeting and you had no intent to act on them) or in which you acted on suicidal thoughts (e.g., did something to hurt yourself with at least some intent to die). I realize that talking about this can be difficult and can bring up concerns about confidentiality, so I’d like to pause for a moment to emphasize that these questions are voluntary and to reiterate what we talked about earlier [during informed consent] about confidentiality [that your responses will not be reported to others, including VA healthcare providers, family, or any outside my research team, unless you reveal that you are currently planning to hurt yourself or planning to do so in the near future. Any questions?] Please take a moment to think about if you have ever had such experiences (thoughts of suicide or suicidal behaviors, in which you took any steps to act on those thoughts]. If so, can you please briefly tell me about this, such as when this occurred, what happened prior to this, and what happened afterwards.

1. Looking back, what precipitated those thoughts or behaviors for you? Were there any warning signs?
2. What did you find to be helpful, if anything, when you were experiencing such thoughts?
   - For example, reaching out to others, specific sources of support (e.g., people, places), things that you told yourself, or ways of coping?
3. Looking back, what would have been helpful to you at that time? What messages would have been helpful for coping with those thoughts and who would be best suited to deliver those messages?
4. When you were having those thoughts, did you consider disclosing to anyone that you were having such thoughts?
   - If not, please tell me more about why not.
   - If you disclosed to someone that you were thinking about suicide, who did you share that with and how did they respond?
5. Did you disclose those thoughts to any healthcare professionals, such as a mental health provider, crisis line worker, etc.? If so, please describe what that was like for you. If not, please describe why not. If you had disclosed this, how would you want them to respond?

**Help-seeking and healthcare use and experiences**

1. Please tell me a little bit about any mental health treatment or suicide prevention treatment you have received (e.g., what this was; whether VA or non-VA) or times you have used crisis services.
   1. What was that like for you?
   2. How has being an AANHPI Veteran impacted your beliefs and behaviors related to seeking help for mental health or emotional distress or suicide?
2. To what extent have your healthcare providers asked you about your culture/race/ethnicity?
   1. What was that like for you (or what would that be like for you)?
3. Have your healthcare providers included any aspects of your culture in personalizing your treatment/healthcare?
   1. What was that like (or what would that be like) for you?

**Safety Planning**

1. Have you ever received a Safety Plan, also known as a Crisis Response Plan?
   1. If so, what was that like for you?
   2. Were any aspects particularly helpful or unhelpful?
   3. Is there anything that you think healthcare providers should take into account when conducting Safety Plans with AANHPI Veterans?

**Caring Contacts**

1. Have you ever received a message, such as a letter, from a VA provider or Suicide Prevention Coordinator thanking you for reaching out and letting you know that they cared about you?
   1. If so, what was that like for you? Were any aspects particularly helpful or unhelpful?
   2. If not, what would that be like for you? Is there anything that you think healthcare providers should consider when sending such messages to AANHPI Veterans?
   3. Who would you prefer to send such messages, if anyone, and what should such messages entail when sending them to AANHPI Veterans?
   4. Are there any important considerations for sending such messages to AANHPI Veterans?

**Concluding remarks**

20. Is there anything else you’d like to share about to how to prevent suicide among Asian and/or Pacific Islander Veterans?
